# Supplementary material for: Ca²⁺ leakage is a conserved signal for non-canonical ATG8/LC3 lipidation and membrane repair
Source: EMBO J. 2026 Mar 20;45(9):3022–55. doi: 10.1038/s44318-026-00741-z (PMC13144738; doi:10.1038/s44318-026-00741-z)
Supplement: Supplementary file 2 — Movie EV1 [file 44318_2026_741_MOESM2_ESM.zip › Movie EV1.docx]

**Movie EV1:** **LLOMe treatment induces lysosome Ca^2+^ leakage.** THP-1 macrophages stably expressing LAMP1-GCaMP6f (green) were treated with LLOMe and imaged by live-cell confocal microscopy at 7-second intervals. Time 0 corresponds to the frame acquired at the start of LLOMe treatment, after a 15-minute stabilization period. Images were processed using Gaussian blur with a sigma (radius) of 1.
