# Supplementary material for: Ca²⁺ leakage is a conserved signal for non-canonical ATG8/LC3 lipidation and membrane repair
Source: EMBO J. 2026 Mar 20;45(9):3022–55. doi: 10.1038/s44318-026-00741-z (PMC13144738; doi:10.1038/s44318-026-00741-z)
Supplement: Supplementary file 3 — Movie EV2 [file 44318_2026_741_MOESM3_ESM.zip › Movie EV2.docx]

**Movie EV2:** **LLOMe treatment induces LC3 conjugation to membranes**. THP-1 macrophages stably expressing RFP (red)-GFP (green)-LC3B were treated with LLOMe and imaged by live-cell confocal microscopy at 25-second intervals. Time 0 corresponds to the frame acquired at the start of LLOMe treatment, after a 5-minute stabilization period. Images were processed using Gaussian blur with a sigma (radius) of 1.
