# Supplementary material for: Ca²⁺ leakage is a conserved signal for non-canonical ATG8/LC3 lipidation and membrane repair
Source: EMBO J. 2026 Mar 20;45(9):3022–55. doi: 10.1038/s44318-026-00741-z (PMC13144738; doi:10.1038/s44318-026-00741-z)
Supplement: Supplementary file 6 — Movie EV5 [file 44318_2026_741_MOESM6_ESM.zip › Movie EV5.docx]

**Movie EV5: STED super resolution imaging reveals the dynamic of LC3-TVS during LLOMe treatment.** THP-1 macrophages stably expressing Halo-LC3B were labelled with Halo dye (white) and subjected to LLOMe treatment. Cells were imaged using STED super-resolution microscopy at 15-second intervals. Time 0 denotes the frame captured at the onset of LLOMe treatment, following a 10-minute stabilization period.
