# Supplementary material for: Ca²⁺ leakage is a conserved signal for non-canonical ATG8/LC3 lipidation and membrane repair
Source: EMBO J. 2026 Mar 20;45(9):3022–55. doi: 10.1038/s44318-026-00741-z (PMC13144738; doi:10.1038/s44318-026-00741-z)
Supplement: Supplementary file 7 — Movie EV6 [file 44318_2026_741_MOESM7_ESM.zip › Movie EV6.docx]

**Movie EV6: VT-iSIM super resolution imaging reveals the dynamic of LC3-TVS during LLOMe treatment.** THP-1 macrophages stably expressing GFP-LC3B were treated with LLOMe. After a 10-minute incubation/stabilization period, time 0 was defined as the first frame acquired and imaging proceeded at 15-second intervals using VT-iSIM super-resolution microscopy. Z-stacks spanning ~5 µm (0.5 µm step size) were collected to cover nearly the entire cell volume, and maximum-intensity projections are shown. Image processing and deconvolution were performed with Huygens Essential (Scientific Volume Imaging).
