# Supplementary material for: Ca²⁺ leakage is a conserved signal for non-canonical ATG8/LC3 lipidation and membrane repair
Source: EMBO J. 2026 Mar 20;45(9):3022–55. doi: 10.1038/s44318-026-00741-z (PMC13144738; doi:10.1038/s44318-026-00741-z)
Supplement: Supplementary file 8 — Movie EV7 [file 44318_2026_741_MOESM8_ESM.zip › Movie EV7.docx]

**Movie EV7: Correlative Array tomography reveals complex LC3-TVS after LLOMe treatment at the nanoscale.** Correlative volume EM image showing the alignment of RFP-GFP-LC3B (yellow) fluorescence with a reconstructed array tomography SEM of a THP-1 macrophage after LLOMe treatment. Membranes were segmented (blue and purple) to show their relationship in 3D.
