# Supplementary material for: Ca²⁺ leakage is a conserved signal for non-canonical ATG8/LC3 lipidation and membrane repair
Source: EMBO J. 2026 Mar 20;45(9):3022–55. doi: 10.1038/s44318-026-00741-z (PMC13144738; doi:10.1038/s44318-026-00741-z)
Supplement: Supplementary file 9 — Movie EV8 [file 44318_2026_741_MOESM9_ESM.zip › Movie EV8.docx]

**Movie EV8: The complex multimembrane morphology of LC3-TVS associated with lipofectamine-coated beads was visualized using high-pressure freezing and AT-SEM.** A Z-stack AT-SEM reconstruction reveals complex multimembrane structures enclosing lipofectamine-coated beads, which are RFP⁺/GFP⁺ and LC3-positive, as shown in Figure 4E. The bead itself was largely dissolved during freeze-substitution, leaving only a small, shrunken remnant visible in the movie. The remaining space left by the bead is not perfectly circular due to section compression. By extrapolation, other visible structures in this dataset are likely compressed to a similar extent—approximately 30% in the plane of sectioning(Studer & Gnaegi, 2000).
