# Supplementary material for: Ca²⁺ leakage is a conserved signal for non-canonical ATG8/LC3 lipidation and membrane repair
Source: EMBO J. 2026 Mar 20;45(9):3022–55. doi: 10.1038/s44318-026-00741-z (PMC13144738; doi:10.1038/s44318-026-00741-z)
Supplement: Supplementary file 16 — Expanded View Figures [file 44318_2026_741_MOESM16_ESM.pdf]

Expanded View Figures

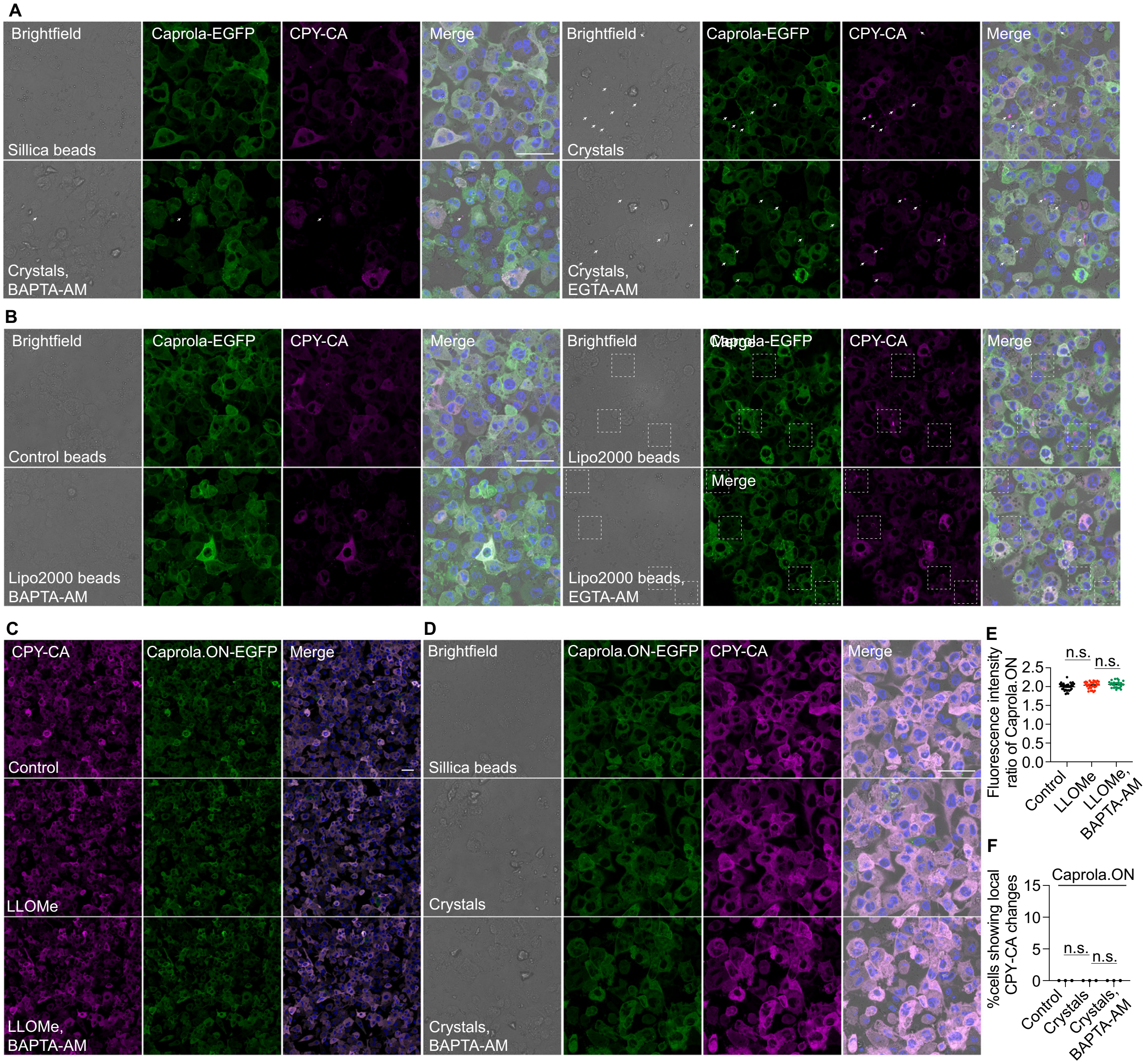

Figure EV1. CPY-CA labelling of Caprola after endomembrane damage depends on Ca^{2+} leakage.

(**A**) CPY-CA staining in THP-1 cells stably expressing Caprola-GFP following phagocytosis of silica crystals or silica beads under the indicated conditions. Arrow heads indicate cells showing Crystal-phagosomal Ca^{2+} leakage. (**B**) CPY-CA staining in THP-1 cells stably expressing Caprola-GFP following phagocytosis of lipofectamine-coated or control beads under the indicated conditions. Dash square indicates cells showing bead-phagosomal Ca^{2+} leakage. (**C**) CPY-CA staining in THP-1 cells stably expressing Caprola-2.ON-GFP under the indicated treatments. (**D**) CPY-CA staining in THP-1 cells stably expressing Caprola-2.ON-GFP following phagocytosis of silica crystals or silica beads under the indicated conditions. (**E**) Quantification of the CPY-CA/EGFP fluorescence intensity ratio corresponding to (**C**). Data points represent individual cells ($n = 30$) from three independent experiments. (**F**) Quantification of the percentage of cells exhibiting crystals/beads uptake exhibiting local CPY-CA changes, defined by changes in the CPY-CA/EGFP fluorescence intensity ratio surrounding bead/crystal phagosomes (related to (**D**)). Data from three independent experiments, with >141 cells analysed per condition. Scale bar: (**A**–**D**), 50 µm.

© The Author(s)

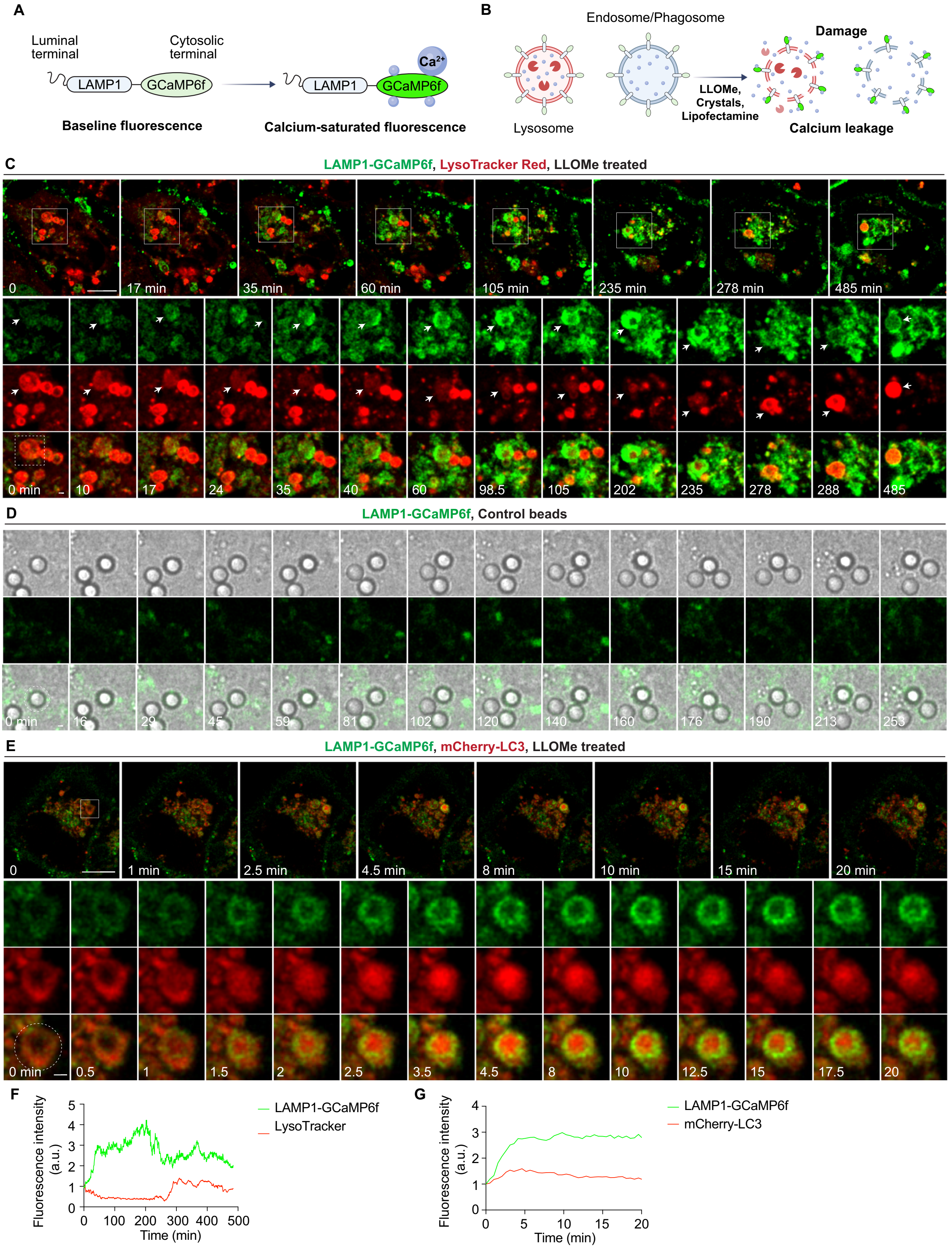
A
Luminal terminal
Cytosolic terminal
LAMP1
GCaMP6f
Ca2+
Baseline fluorescence
Calcium-saturated fluorescence
B
Endosome/Phagosome
Damage
LLOMe, Crystals, Lipofectamine
Lysosome
Calcium leakage
C
LAMP1-GCaMP6f, LysoTracker Red, LLOMe treated
0
17 min
35 min
60 min
105 min
235 min
278 min
485 min
0 min
10
17
24
35
40
60
98.5
105
202
235
278
288
485
D
LAMP1-GCaMP6f, Control beads
0 min
16
29
45
59
81
102
120
140
160
176
190
213
253
E
LAMP1-GCaMP6f, mCherry-LC3, LLOMe treated
0
1 min
2.5 min
4.5 min
8 min
10 min
15 min
20 min
0 min
0.5
1
1.5
2
2.5
3.5
4.5
8
10
12.5
15
17.5
20
F
Fluorescence intensity (a.u.)
Time (min)
LAMP1-GCaMP6f
LysoTracker
G
Fluorescence intensity (a.u.)
Time (min)
LAMP1-GCaMP6f
mCherry-LC3

 © The Author(s)

Figure EV2. Endomembrane damage induces Ca^{2+} leakage and initiates LC3 lipidation.

(**A**) Schematic representation of the LAMP1-GCaMP6f reporter. GCaMP6f is localized to the cytosolic side of human LAMP1 and exhibits baseline fluorescence under resting conditions. Upon Ca^{2+} binding, the fluorescence intensity of GCaMP6f increases significantly. (**B**) Diagram illustrating the localization of LAMP1-GCaMP6f to phagosomes, where it maintains baseline fluorescence. During endolysosomal/phagosomal damage, Ca^{2+} leaks into the cytosol and binds to LAMP1-GCaMP6f, resulting in a marked increase in fluorescence intensity. (**C**) Live-cell imaging sequence showing dynamic changes in lysosomal Ca^{2+} leakage (via LAMP1-GCaMP6f) and LysoTracker Red signal during LLOMe treatment. White squares indicate magnified regions. Arrow heads indicate the lysosome underwent membrane damage and repair. The dashed square represents the area used for LAMP1-GCaMP6f and LysoTracker signal quantification in Fig. EV2F. Images were processed using a Gaussian blur with a sigma (radius) of 1. (**D**) Live-cell imaging sequence showing dynamic changes in Ca^{2+} levels surrounding endolysosomes and phagosomes in THP-1 macrophages stably expressing LAMP1-GCaMP6f during phagocytosis of control beads. Dashed circle indicates the bead region used for LAMP1-GCaMP6f signal quantification in Fig. 2F. Images were processed using Gaussian blur with a sigma (radius) of 1. (**E**) Live-cell imaging sequence showing lysosomal Ca^{2+} leakage (LAMP1-GCaMP6f) and mCherry-LC3 signal during LLOMe treatment. White squares indicate magnified regions. The dashed cricle represents the area used for LAMP1-GCaMP6f and mCherry-LC3 signal quantification in Fig. EV2G. Images were processed using a Gaussian blur with a sigma (radius) of 1. (**F**) Fluorescence intensity ratio change (F/F0) of LAMP1-GCaMP6f and LysoTracker fluorescence intensity in Fig. EV2C. (**G**) Fluorescence intensity ratio change (F/F0) of LAMP1-GCaMP6f and mCherry-LC3 fluorescence intensity in EV2E. Scale bars: (**C**, **E**), 10 μm (main images), 1 μm (enlarged insets); (**D**), 1 μm.

© The Author(s)

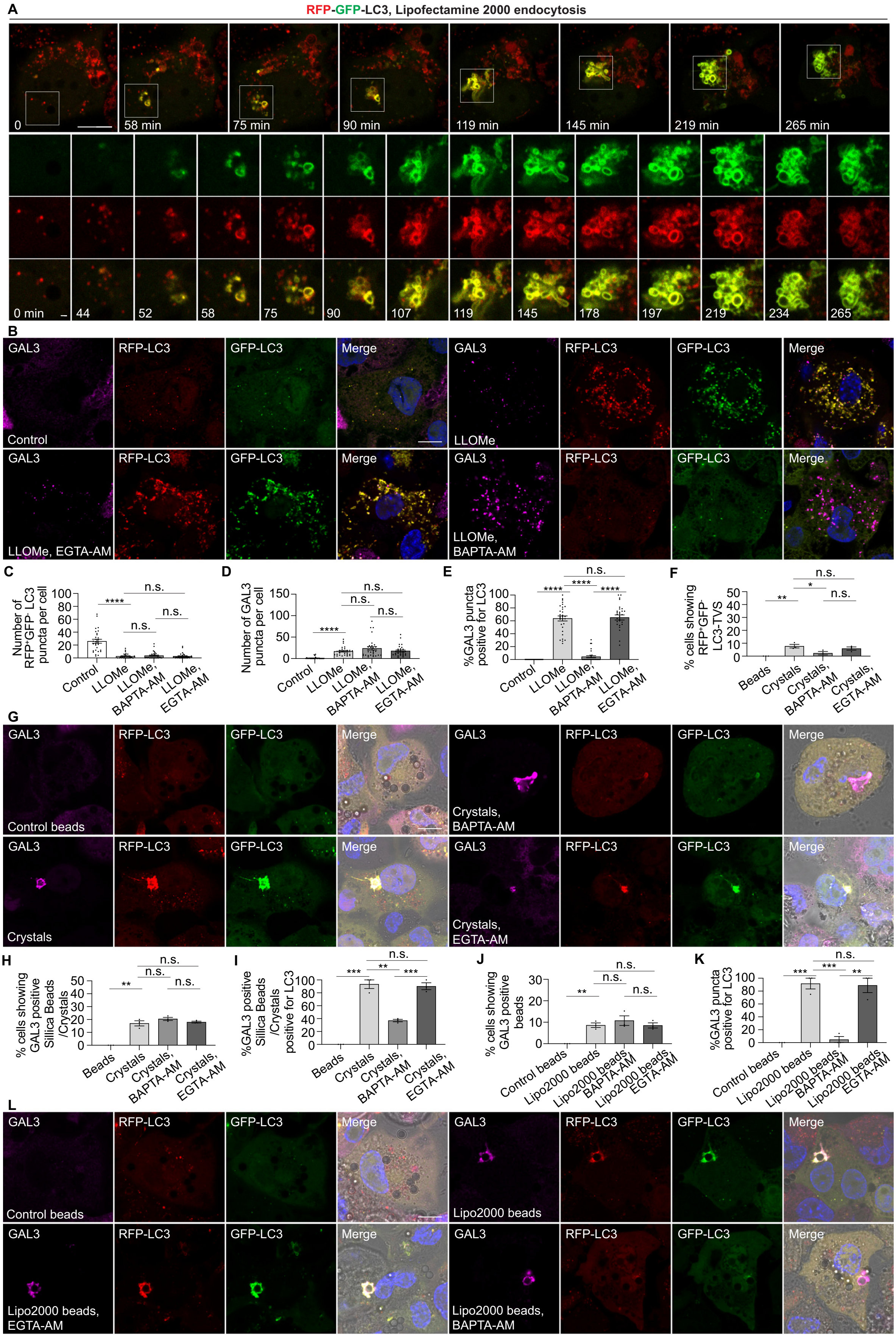
A
RFP-GFP-LC3, Lipofectamine 2000 endocytosis
0
58 min
75 min
90 min
119 min
145 min
219 min
265 min
0 min
44
52
58
75
90
107
119
145
178
197
219
234
265
B
GAL3
RFP-LC3
GFP-LC3
Merge
Control
LLOMe
LLOMe, EGTA-AM
LLOMe, BAPTA-AM
C
Number of RFP+GFP- LC3 puncta per cell
Control
LLOMe
LLOMe, BAPTA-AM
LLOMe, EGTA-AM
D
Number of GAL3 puncta per cell
E
%GAL3 puncta positive for LC3
F
% cells showing RFP+GFP- LC3-TVS
Beads
Crystals
Crystals, BAPTA-AM
Crystals, EGTA-AM
G
Control beads
Crystals, BAPTA-AM
Crystals
Crystals, EGTA-AM
H
% cells showing GAL3 positive Sillica Beads /Crystals
I
%GAL3 positive Sillica Beads /Crystals positive for LC3
J
% cells showing GAL3 positive beads
Control beads
Lipo2000 beads
Lipo2000 beads, BAPTA-AM
Lipo2000 beads, EGTA-AM
K
%GAL3 puncta positive for LC3
L
Control beads
Lipo2000 beads
Lipo2000 beads, EGTA-AM
Lipo2000 beads, BAPTA-AM

 © The Author(s)

◀ **Figure EV3. Sterile endomembrane damage triggers LC3-TVS formation.**

(**A**) Live-cell imaging sequence showing dynamic changes in RFP-GFP-LC3B during treatment with Lipofectamine 2000. White boxes indicate the zoomed-in areas. Images were processed using Gaussian blur with a sigma (radius) of 1. (**B**) GAL3 immunostaining in THP-1 macrophages stably expressing RFP-GFP-LC3B following the indicated treatments. (**C**) Quantification of the number of LC3 puncta positive for RFP but negative for GFP per cell, corresponding to (**B**). A total of 30 cells were analysed from 3 independent experiments. (**D**) Quantification of the number of GAL3-positive puncta per cell, corresponding to (**B**). A total of 30 cells were analysed from 3 independent experiments. (**E**) Quantification of the percentage of GAL3-positive puncta that are also positive for LC3 per cell, corresponding to (**B**). A total of 30 cells were analysed from 3 independent experiments. (**F**) Quantification of the percentage of cells exhibiting crystals/beads uptake displaying bead- or crystal-associated LC3-TVS that are positive RFP but negative for GFP, corresponding to (**G**). Data represent individual experiments; more than 132 cells were analysed per condition. (**G**) GAL3 staining in THP-1 macrophages stably expressing RFP-GFP-LC3B following phagocytosis of crystals or silica beads under the indicated treatments. (**H**) Quantification of the percentage of cells exhibiting crystals/beads uptake containing GAL3-positive beads or crystals, corresponding to (**G**). Data represent individual experiments; more than 132 cells were analysed per condition. (**I**) Quantification of the percentage of GAL3-positive beads or crystals that are also positive for LC3, corresponding to (**G**). Data represent individual experiments; more than 132 cells were analysed per condition. (**J**) Quantification of the percentage of cells exhibiting beads uptake containing GAL3-positive beads, corresponding to (**L**). Data represent individual experiments; more than 119 cells were analysed per condition. (**K**) Quantification of the percentage of GAL3-positive beads that are also positive for LC3, corresponding to (**L**). Data represent individual experiments; more than 119 cells were analysed per condition.
(**L**) Immunofluorescence staining of GAL3 in THP-1 macrophages stably expressing RFP-GFP-LC3B following the phagocytosis of control beads or Lipofectamine 2000-coated beads under the indicated treatments. Scale bars: (**A**), 10 μm (main panels), 1 μm (zoomed-in insets); (**B**, **G**, **L**), 10 μm.

© The Author(s)

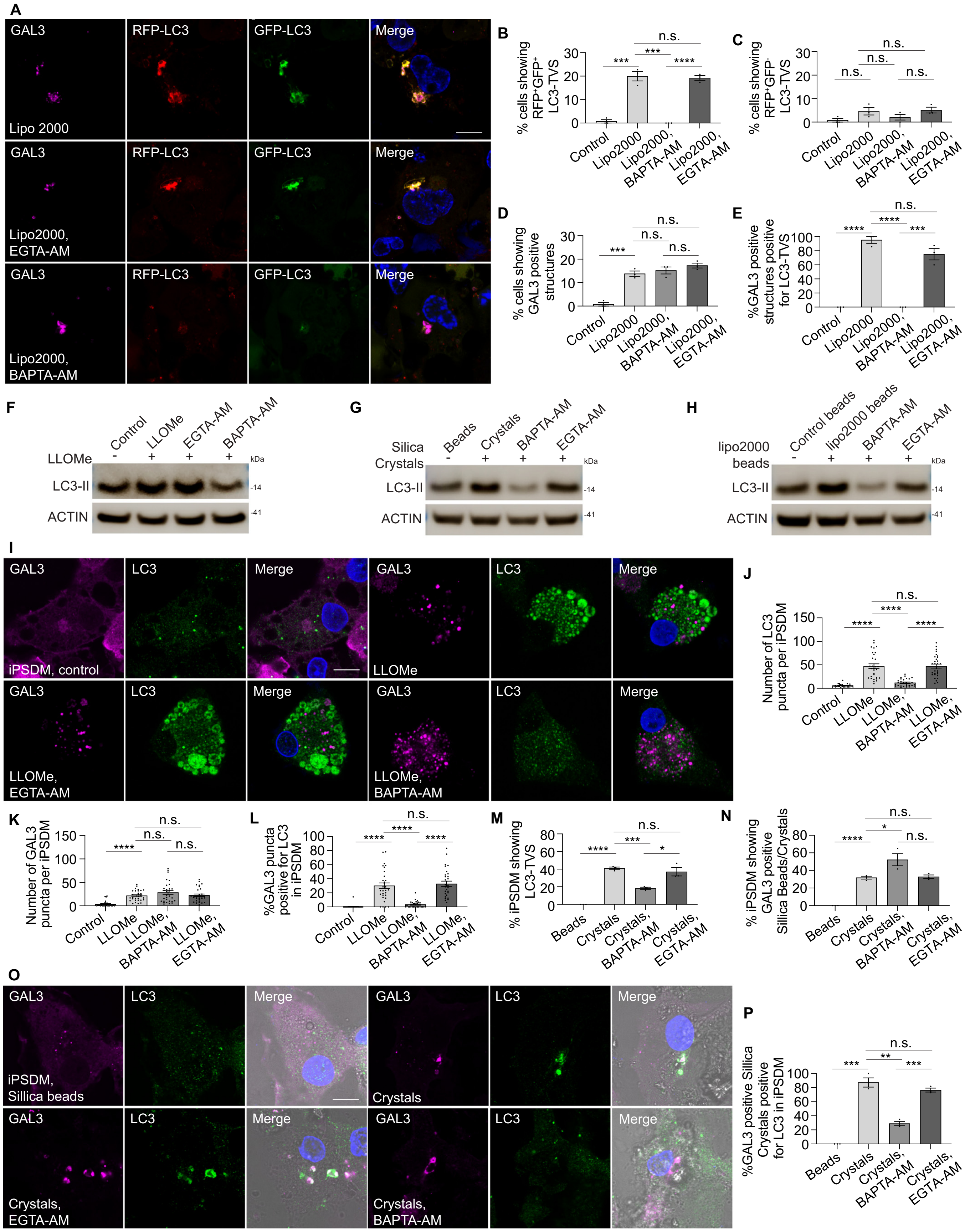
A
GAL3
RFP-LC3
GFP-LC3
Merge
Lipo 2000
Lipo2000, EGTA-AM
Lipo2000, BAPTA-AM
B
% cells showing RFP+GFP+ LC3-TVS
Control
Lipo2000
Lipo2000, BAPTA-AM
Lipo2000, EGTA-AM
C
% cells showing RFP+GFP- LC3-TVS
D
% cells showing GAL3 positive structures
E
%GAL3 positive structures positive for LC3-TVS
F
LLOMe
LC3-II
ACTIN
EGTA-AM
BAPTA-AM
kDa
G
Silica Crystals
Beads
Crystals
H
lipo2000 beads
Control beads
lipo2000 beads
I
iPSDM, control
LLOMe
LLOMe, EGTA-AM
LLOMe, BAPTA-AM
LC3
J
Number of LC3 puncta per iPSDM
K
Number of GAL3 puncta per iPSDM
L
%GAL3 puncta positive for LC3 in iPSDM
M
% iPSDM showing LC3-TVS
N
% iPSDM showing GAL3 positive Sillica Beads/Crystals
O
iPSDM, Sillica beads
Crystals
Crystals, EGTA-AM
Crystals, BAPTA-AM
P
%GAL3 positive Sillica Crystals positive for LC3 in iPSDM
n.s.

Figure EV4. Sterile endomembrane damage triggers LC3-TVS formation in iPSDM.

(**A**) GAL3 immunostaining in THP-1 macrophages stably expressing RFP-GFP-LC3B following the indicated treatments. (**B**) Quantification of the percentage of cells exhibiting LC3-TVS structures that are positive for both RFP-LC3 and GFP-LC3, corresponding to (**A**). Data points represent individual experiments; more than 137 cells were analysed per condition. (**C**) Quantification of the percentage of cells displaying LC3-TVS structures that are positive for RFP-LC3 but negative for GFP-LC3, corresponding to (**A**). Data points represent individual experiments; more than 137 cells were analysed per condition. (**D**) Quantification of the percentage of cells containing GAL3-positive structures, corresponding to (**A**). Data points represent individual experiments; more than 137 cells were analysed per condition.
(**E**) Quantification of the percentage of GAL3-positive structures that also show LC3 signal, corresponding to (**A**). Data points represent individual experiments; more than 137 structures were analysed per condition. (**F–H**) Immunoblotting results showing the LC3 lipidation under indicated conditions. (**I**) Immunostaining of GAL3 and LC3 in iPSC-derived macrophages (iPSDM) following the indicated treatments. (**J**) Quantification of the number of LC3-positive puncta per iPSDM, corresponding to (**I**). $n = 30$ cells from three independent experiments. (**K**) Quantification of the number of GAL3-positive puncta per iPSDM, corresponding to (**I**). $n = 30$ cells from three independent experiments. (**L**) Quantification of the percentage of GAL3-positive puncta that positive for LC3 per iPSDM. $n = 30$ cells from three independent experiments.
(**M**) Quantification of the percentage of iPSDMs exhibiting crystals/beads uptake exhibiting LC3-TVS structures during phagocytosis of silica beads or crystals under the indicated treatments, corresponding to (**O**). Data points represent individual experiments; more than 92 cells were analysed per condition. (**N**) Quantification of the exhibiting crystals/beads uptake of iPSDMs containing GAL3-positive beads or crystals under the indicated treatments, corresponding to (**O**). Data points represent individual experiments; more than 92 cells were analysed per condition. (**O**) Immunostaining of GAL3 and LC3 in iPSDMs following phagocytosis of silica beads or crystals under the indicated treatments. (**P**) Quantification of the percentage of GAL3-positive beads or crystals that are also positive for LC3, corresponding to (**O**). Data points represent individual experiments; more than 92 cells were analysed per condition. Scale bars: (**A**, **I**, **O**), 10 µm.

© The Author(s)

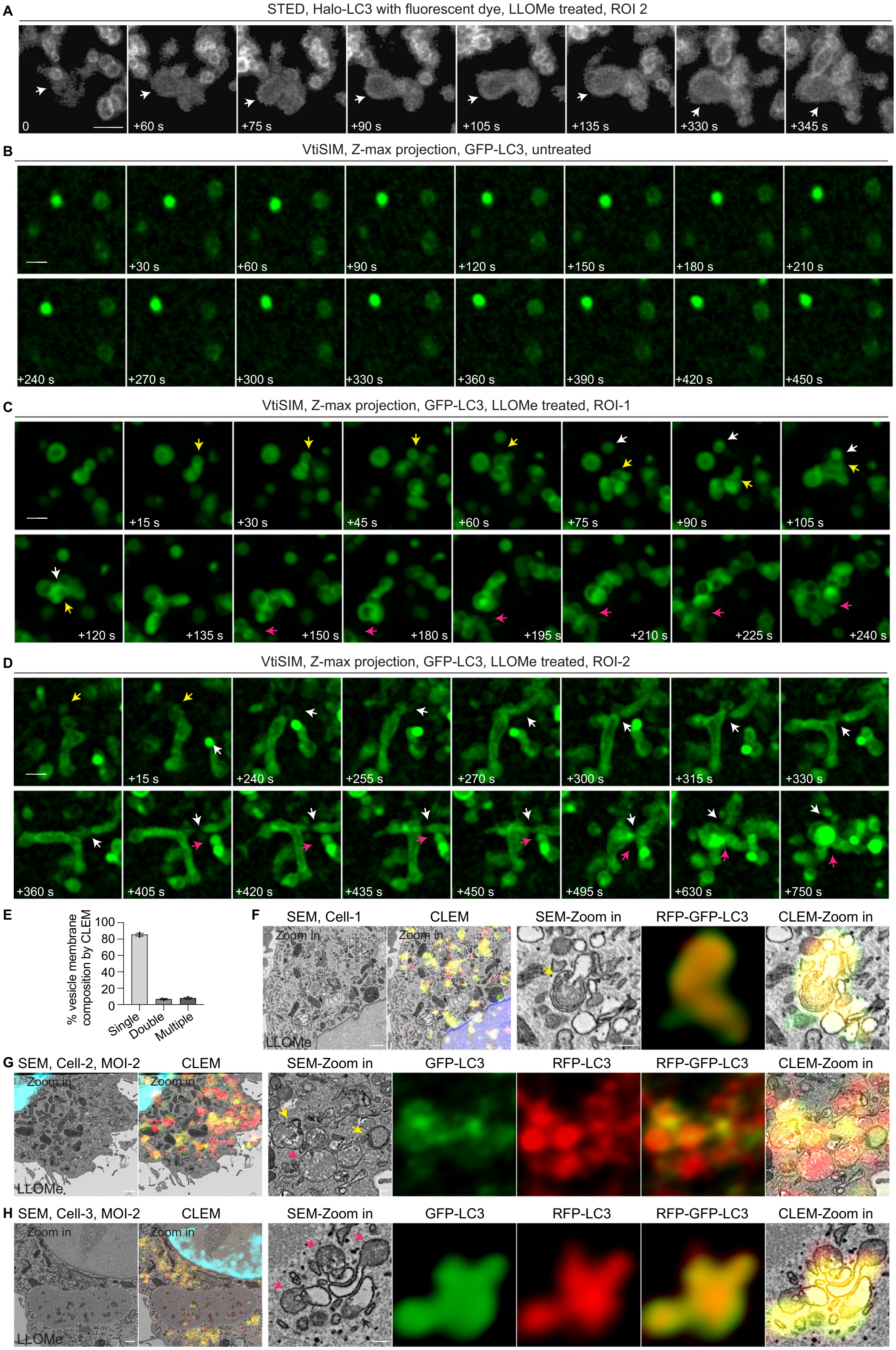
A
STED, Halo-LC3 with fluorescent dye, LLOMe treated, ROI 2
0
+60 s
+75 s
+90 s
+105 s
+135 s
+330 s
+345 s
B
VtiSIM, Z-max projection, GFP-LC3, untreated
+30 s
+60 s
+90 s
+120 s
+150 s
+180 s
+210 s
+240 s
+270 s
+300 s
+330 s
+360 s
+390 s
+420 s
+450 s
C
VtiSIM, Z-max projection, GFP-LC3, LLOMe treated, ROI-1
+15 s
+30 s
+45 s
+60 s
+75 s
+90 s
+105 s
+120 s
+135 s
+150 s
+180 s
+195 s
+210 s
+225 s
+240 s
D
VtiSIM, Z-max projection, GFP-LC3, LLOMe treated, ROI-2
+15 s
+240 s
+255 s
+270 s
+300 s
+315 s
+330 s
+360 s
+405 s
+420 s
+435 s
+450 s
+495 s
+630 s
+750 s
E
% vesicle membrane composition by CLEM
100
80
60
40
20
0
Single
Double
Multiple
F
SEM, Cell-1
CLEM
SEM-Zoom in
RFP-GFP-LC3
CLEM-Zoom in
Zoom in
LLOMe
G
SEM, Cell-2, MOI-2
CLEM
SEM-Zoom in
GFP-LC3
RFP-LC3
RFP-GFP-LC3
CLEM-Zoom in
Zoom in
LLOMe
H
SEM, Cell-3, MOI-2
CLEM
SEM-Zoom in
GFP-LC3
RFP-LC3
RFP-GFP-LC3
CLEM-Zoom in
Zoom in
LLOMe

 © The Author(s)

Figure EV5. Super resolution imaging and CLEM reveal LC3-positive membranes induced by endomembrane damage are highly dynamic multilamellar structures.

(**A**) STED super-resolution imaging of THP-1 macrophages stably expressing Halo-LC3, labelled with Halo dye, under 10 min LLOMe treatment. White arrowheads indicate the LC3-TVS underwent dynamic fusion. (**B–D**) Time-lapse images showing membrane remodelling of LC3-positive vesicles induced by lysosomal damage. THP-1 macrophages stably expressing GFP-LC3B were under normal condition or treated with LLOMe. Cells were imaged using VT-iSIM super-resolution microscopy at 15-second intervals. Images are z-maximum projections covering entire cells (0.5 µm per stack, 5–7 µm total). Time 0 indicates the frame acquired at the onset of LLOMe treatment, following a 10-min stabilization period. LC3-positive structures under untreated conditions (**B**). Representative regions of interest (ROIs) showing dynamic LC3-positive vesicles from three independent experiments (related to Movie EV6) under LLOMe treatment (**C**, **D**). Yellow, pink, and white arrows indicate distinct docking and fusion events observed over time. (**E**) Quantification of percentage of single-, double-, and multiple-membrane RFP^+GFP^+ LC3-positive vesicles relative to the total number of RFP^+GFP^+ LC3-positive vesicles determined by CLEM ($n = 3$ cells, 167 vesicles were analysed), related to Fig. 4B–D and F–H. (**F**) CLEM analysis showing RFP-LC3 and GFP-LC3 double-positive multimembrane structures following LLOMe treatment (related to Fig. 4B and Movie EV7). Yellow arrowhead indicates the LC3-positive multimembranes. (**G**, **H**) CLEM analysis reveals complex membrane structures positive for both RFP-LC3 and GFP-LC3 after LLOMe treatment. Yellow arrowheads indicate the LC3-positive multimembranes. Pink arrowheads indicate the LC3-positive single membrane. Scale bars: (**A–D**), 1 µm; (**F–H**), 1 µm (main images), 200 nm (zoomed-in areas).

© The Author(s)

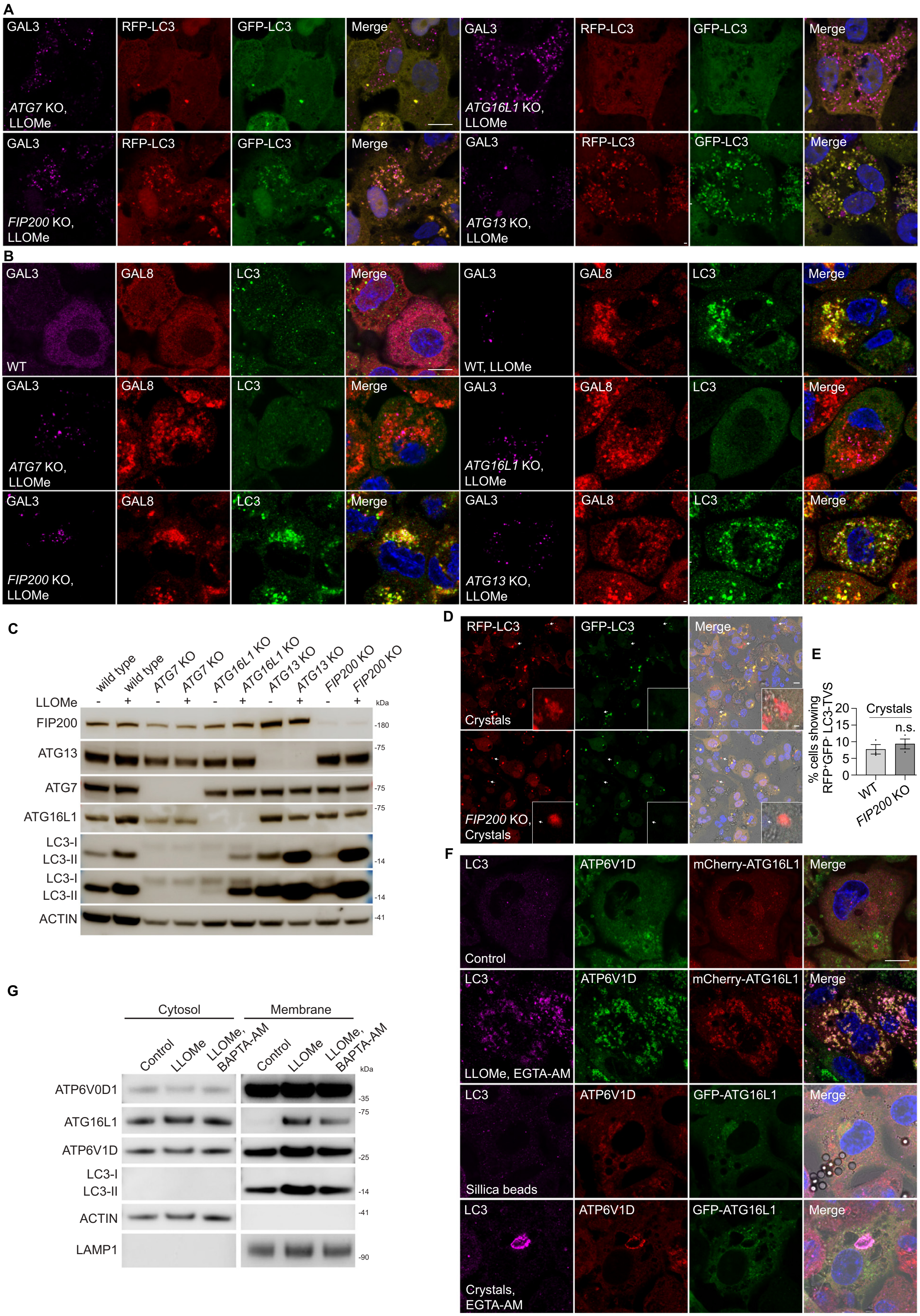
A
GAL3
RFP-LC3
GFP-LC3
Merge
ATG7 KO, LLOMe
ATG16L1 KO, LLOMe
FIP200 KO, LLOMe
ATG13 KO, LLOMe
B
GAL3
GAL8
LC3
Merge
WT
WT, LLOMe
ATG7 KO, LLOMe
ATG16L1 KO, LLOMe
FIP200 KO, LLOMe
ATG13 KO, LLOMe
C
wild type
wild type
ATG7 KO
ATG7 KO
ATG16L1 KO
ATG16L1 KO
ATG13 KO
ATG13 KO
FIP200 KO
FIP200 KO
LLOMe
kDa
FIP200
ATG13
ATG7
ATG16L1
LC3-I
LC3-II
LC3-I
LC3-II
ACTIN
-180
-75
-75
-75
-14
-14
-41
D
RFP-LC3
GFP-LC3
Merge
Crystals
FIP200 KO, Crystals
E
Crystals
n.s.
% cells showing RFP+GFP- LC3-TVS
20
15
10
5
0
WT
FIP200 KO
F
LC3
ATP6V1D
mCherry-ATG16L1
GFP-ATG16L1
Merge
Control
LLOMe, EGTA-AM
Sillica beads
Crystals, EGTA-AM
G
Cytosol
Membrane
Control
LLOMe
LLOMe, BAPTA-AM
kDa
ATP6V0D1
ATG16L1
ATP6V1D
LC3-I
LC3-II
ACTIN
LAMP1
-35
-75
-25
-14
-41
-90

© The Author(s)

Figure EV6. LC3-TVS formation is independent of canonical autophagy and requires the V-ATPase-ATG16L1 complex.

(**A**) GAL3 staining in WT, *ATG16L1* KO, *FIP200* KO, *ATG13* KO, and *ATG7* KO THP-1 macrophages stably expressing RFP-GFP-LC3B after LLOMe treatment, related to Fig. 5D,E. (**B**) GAL3, GAL8 and LC3 staining in WT, *ATG16L1* KO, *FIP200* KO, *ATG13* KO, and *ATG7* KO THP-1 macrophages after LLOMe treatment, related to Fig. 5I,J. (**C**) Immunoblotting results showing LC3 lipidation under LLOMe-induced membrane damage in the indicated autophagy mutant cells. (**D**) RFP-GFP-LC3B stably expressed WT and *FIP200* KO THP-1 macrophages after 3 h Silica Crystals phagocytosis. (**E**) Quantification shows the percentage of cells showing $RFP^{+}GFP^{-}$ Crystal-LC3-TVS; 144 and 146 cells were analysed from three independent experiments, related to (**D**). (**F**) LC3 and ATP6V1D staining in THP-1 macrophages stably expressing mCherry-ATG16L1 or GFP-ATG16L1 under indicated treatment, related to Fig. 6F–K. (**G**) Subcellular fractionation assay showing the recruitment of the indicated proteins to the membrane. ACTIN, as well as the membrane proteins ATP6V0D1, were used as controls. Scale bars: (**A**, **B**, **F**), 10 µm, (**D**), 10 µm (main panels), 1 µm (zoomed-in insets).

© The Author(s)

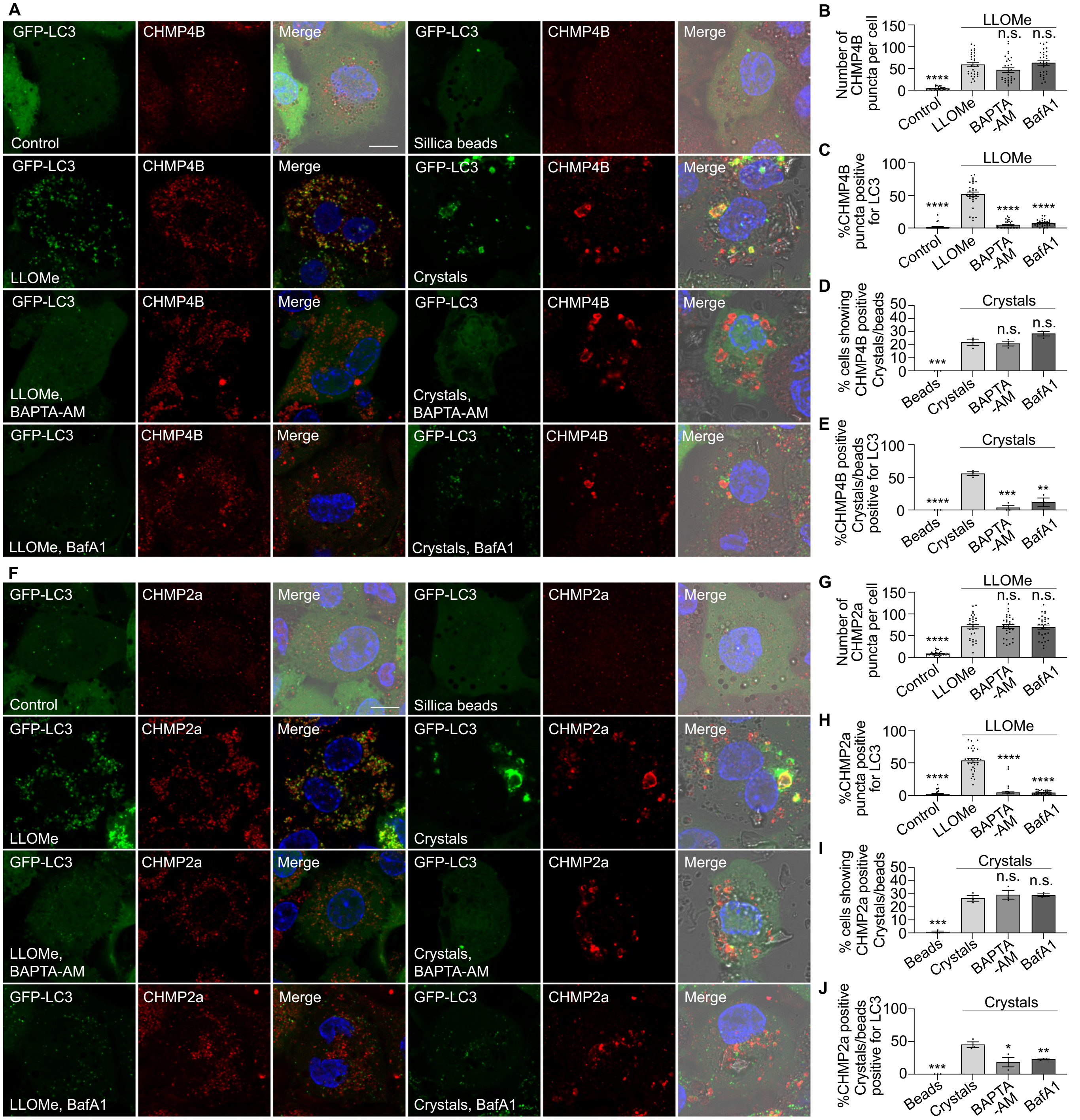
A
GFP-LC3
CHMP4B
Merge
Control
Sillica beads
LLOMe
Crystals
LLOMe,
BAPTA-AM
Crystals,
BAPTA-AM
LLOMe, BafA1
Crystals, BafA1
B
Number of CHMP4B puncta per cell
LLOMe
n.s.
****
Control
BAPTA -AM
BafA1
C
%CHMP4B puncta positive for LC3
D
% cells showing CHMP4B positive Crystals/beads
Beads
E
%CHMP4B positive Crystals/beads positive for LC3
F
CHMP2a
G
Number of CHMP2a puncta per cell
H
%CHMP2a puncta positive for LC3
I
% cells showing CHMP2a positive Crystals/beads
J
%CHMP2a positive Crystals/beads positive for LC3

 © The Author(s)

Figure EV7. LC3-TVS-mediated membrane repair is independent of the recruitment of the ESCRT machinery.

(**A**) Immunostaining of CHMP4B in THP-1 macrophages stably expressing GFP-LC3B under the indicated treatments. (**B**) Quantification of CHMP4B-positive puncta per cell, corresponding to (**A**). $n = 30$ cells from three independent experiments. (**C**) Quantification of the percentage of CHMP4B-positive puncta that are also LC3-positive per cell, corresponding to (**A**). $n = 30$ cells from three independent experiments. (**D**) Quantification of the cells exhibiting crystals/beads uptake containing CHMP4B-positive beads or crystals, corresponding to (**A**). Data points represent individual experiments; more than 144 cells were analysed per condition. (**E**) Quantification of the percentage of CHMP4B-positive beads or crystals that are also LC3-positive, corresponding to (**A**). Data points represent individual experiments; more than 144 cells were analysed per condition. (**F**) Immunostaining of CHMP2A in THP-1 macrophages stably expressing GFP-LC3B under the indicated treatments. (**G**) Quantification of CHMP2A-positive puncta per cell, corresponding to (**F**). $n = 30$ cells from three independent experiments. (**H**) Quantification of CHMP2A-positive puncta that are also LC3-positive per cell, corresponding to (**F**). $n = 30$ cells from three independent experiments. (**I**) Quantification of the cells exhibiting crystals/beads uptake containing CHMP2A-positive beads or crystals, corresponding to (**F**). Data points represent individual experiments; more than 138 cells were analysed per condition. (**J**) Quantification of the percentage of CHMP2A-positive beads or crystals that are also LC3-positive, corresponding to (**F**). Data points represent individual experiments; more than 138 structures were analysed per condition. Scale bars: (**A**, **F**) = 10 μm.

© The Author(s)

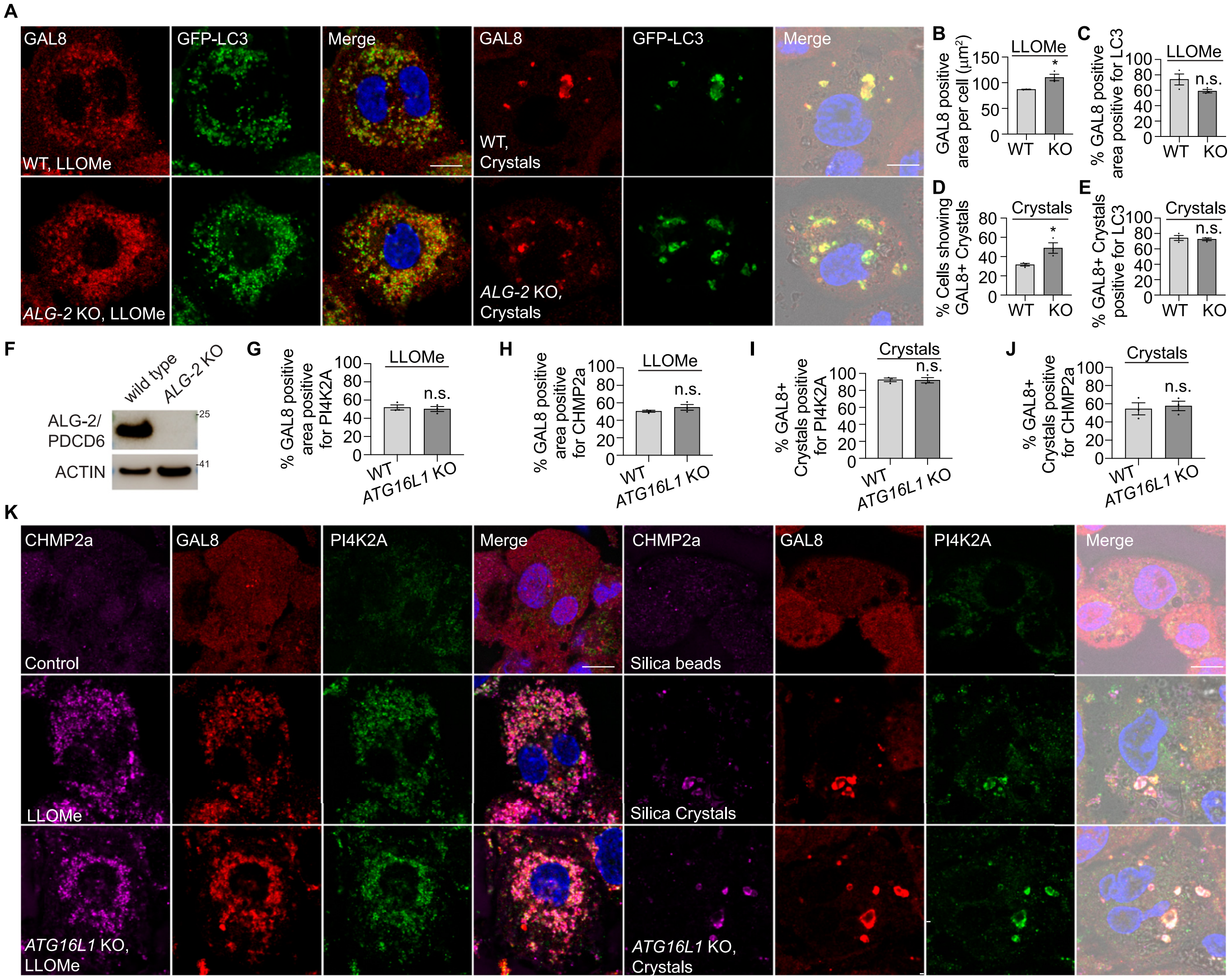

Figure EV8. LC3-TVS-mediated membrane repair is independent of ALG-2 or the recruitment of the PI4K2A.

(**A**) Immunofluorescence staining of GAL8 in GFP-LC3 stably expressing WT and *ALG-2* KO THP-1 macrophages following LLOMe or silica crystal treatment.
(**B**) Quantification of the GAL8-positive area per cell in WT and *ALG-2* KO cells after LLOMe treatment, related to (**A**). A total of 144 (WT) and 145 (KO) cells from three independent experiments were analysed using ImageJ (Analyze Particles). (**C**) Quantification of the percentage of GAL8-positive area colocalized with LC3, related to (**A**). 144 and 145 cells from three experiments were analysed using ImageJ (Image Calculator). (**D**) Quantification of the percentage of cells exhibiting crystals/beads uptake containing GAL8-positive crystal phagosomes, related to (**A**). 158 (WT) and 181 (KO) cells from three independent experiments were analysed. (**E**) Quantification of the percentage of GAL8-positive crystal phagosomes that are also LC3-positive, related to (**A**). 158 and 181 cells from three independent experiments were analysed.
(**F**) Immunoblotting results showing the level of ALG-2 in WT and *ALG-2* KO THP-1 macrophages. (**G**) Quantification of the percentage of GAL8-positive area colocalized with PI4K2A, related to (**K**). A total of 171 (WT) and 193 (KO) cells from three independent experiments were analysed using ImageJ. (**H**) Quantification of the percentage of GAL8-positive area colocalized with CHMP2A, related to (**K**). 171 and 193 cells from three experiments were analysed using ImageJ. (**I**) Quantification of the percentage of GAL8-positive crystal phagosomes that are also PI4K2A-positive, related to (**K**). 190 (WT) and 205 (KO) cells from three independent experiments were analysed. (**J**) Quantification of the percentage of GAL8-positive crystal phagosomes that are also CHMP2A-positive, related to (**K**). 190 and 205 cells from three independent experiments were analysed. (**K**) Immunofluorescence staining of CHMP2A, GAL8 and PI4K2A in WT and ATG16L1 KO THP-1 macrophages following LLOMe or silica crystal treatment. Scale bars: 10 μm.

 © The Author(s)
